# Mini review: Apple improvement, traditional approaches, biotechnology options, and regulatory considerations

**DOI:** 10.3389/fbioe.2025.1617110

**Published:** 2025-06-04

**Authors:** Amy L. Klocko

**Affiliations:** Department of Biology, University of Colorado Colorado Springs, Colorado Springs, CO, United States

**Keywords:** apple, biotechnology, germplast, breeding, GMO, apple scab, fire blight

## Abstract

Apples are a popular and globally important crop. The fruits are eaten fresh, pressed for juice, fermented as cider, processed into sauce, dried, and more. There are thousands of different cultivars, a small subset of which are grown on a commercial scale. Genetic analysis has shown that, as a group, domestic apples have a complicated genetic background, with contributions from multiple wild species. By contrast, most of the highly produced commercialized modern cultivars share a narrow range of genetic diversity. However, as apples are outcrossing, propagated vegetatively, and long-lived, wild and heirloom varieties can be maintained and are valuable sources of genetic diversity for desirable traits. Apples are also amenable to genetic transformation, and work in this area has resulted in improved resistance to diseases and a commercialized non-browning variety, the Arctic™ Apple. Traditional breeding, breeding guided by modern genetic knowledge, and biotechnology all contribute to the overall process of apple cultivar development and represent an important example of how many approaches can be used in crop improvement. As global biosafety regulations continue to develop and change, countries will be tasked with developing guidelines for both the creation and import of apple trees and apple products.

## 1 Introduction

*Malus* x *domestica* Borkh. (domestic apple, hereafter apple) is a staple fruit crop grown in many regions of the world. In 2023, over 661,000 ha of apple trees were harvested, mostly in China, India, and Russia, with over 90 countries reporting output (FAO, 2023).

In general, apples are considered either dessert or cider, with the latter being used for the brewing of fermented alcoholic cider. Dessert apples are intended for the fresh fruit market, and are also processed into juice, vinegar, applesauce, dried fruit, and more (Downing, 1989; Guine et al., 2021). Dessert apple juice can be consumed fresh, or fermented into cider, but this approach is aimed at meeting rising consumer as cider cultivars are less commonly grown than dessert varieties (Soomro et al., 2022). By contrast, traditional cider apples are not meant for fresh consumption and are sometimes referred to as “spitters” due to their high phenolic content and rather unpalatable fruits (Marks et al., 2007).

## 2 Genetics and domestication

Domestic apple was one of the earlier plant genomes to be sequenced, with the first genome version published in 2010 (Velasco et al., 2010). Apples were domesticated in Asia, and genetic analysis of wild and domestic varieties shows evidence of breeding with local wild species in both Asia and Europe as varieties were developed (Cornille et al., 2014; Chen et al., 2023). Many countries have bred locally important varieties of apples, and there is interest in preserving these heritage varieties (see Supplementary Table S1 and examples therein).

Of the over 7,000 domestic apple cultivars, nearly all commercial production is from just a handful of varieties, which share a lot of common genetic heritage (Noiton and Alspach, 1996; Forsline et al., 2003; Pereira-Lorenzo et al., 2018). This lack of diversity in existing elite cultivars adds to the challenge of apple improvement. In general, domestic apple trees are outcrossing, and varieties of interest are maintained by vegetative propagation, often involving grafting a long-established practice (Cornille et al., 2014; Pereira-Lorenzo et al., 2018). As these trees can also be long lived, very old varieties can still be found in modern times. In addition, many wild varieties of *Malus* are compatible with domestic trees, providing an important resource for key traits (Cornille et al., 2014).

### 2.1 Select apple traits of interest

Like crops in general, apple trees are vulnerable to pathogens, which cause significant economic losses. These include the fungus *Venturia inaequalis* which causes apple scab, *Erwinia amylovora*, causing fire blight, *Penicillium expansum* leading to postharvest disease, and more (MacHardy, 1996; Malnoy et al., 2012; Luciano-Rosario et al., 2020). In general, highly produced commercial cultivars exhibit little to no resistance to those three diseases, which are managed through cultivation practices (Gessler et al., 2006; Holb, 2007; Kellerhals et al., 2012; Luciano-Rosario et al., 2020). Similarly, post-harvest losses from fungal growth can be considerable, with little genetic resistance, fungicides re the typical control method (Gupta and Saxena, 2023). There is great interest in identifying or developing disease resistant cultivars.

In addition to disease resistance, interesting marketable fruit traits are sought after, such as red fleshed fruits, tiny fruits, large sized fruits, or early ripening (Janick et al., 1996; Volz et al., 2009). Other preferred characteristics of fresh market apples such as fruit color, pattern, and texture are variable by region and across time (Janick et al., 1996). As apples intended for the fresh fruit market can undergo extensive storage to allow year-round sales of this seasonal fruit, longer shelf-life is a valuable trait (Janick et al., 1996). Consumers are also interested in obtaining nutritious foods (Rahman et al., 2021). Apples are high in a variety of compounds, including ascorbic acid, commonly known as vitamin C (overviewed in (Planchon et al., 2004)). Analysis of vitamin C content of apple indicates both genes and the environment factor into vitamin levels, with older cultivars tending to have much higher levels on average than newer varieties (Planchon et al., 2004).

### 2.2 Approaches to cultivar development

Given the great diversity of apple cultivars as a group and the presence of genetically compatible wild relatives, there are abundant genetic resources available for potentially obtaining apples with desired genetically encoded traits. Apple germplasm collections in countries around the world are valuable resources for conserving and documenting this diversity (Supplementary Table S1). Phenotype screening of collections of wild and domestic apple have identified resistance to fire blight, apple scab, and other diseases, and some individuals even exhibit multiple resistance (Luby et al., 2002; Volk et al., 2008). An addition, extensive research identifying candidate genes or quantitative trait loci (QTLs) for fire blight and apple scab resistance now makes it possible to perform marker assisted seeding selection (MASS) in apple (Ru et al., 2015). Thus, trees and progeny can now be genotyped to track traits genetically. The long juvenile period and lack of self-compatibility of apples in general means is time-consuming to integrate traits and develop cultivars using naturally occurring plant maturation (Pereira-Lorenzo et al., 2018). This approach works well, and breeding efforts started in the 1940s have successfully bred in resistance to apple scab from wild *M. floribunda* into domestic apple (Crosby et al., 1992). Similar efforts to create additional scab-resistant apple cultivars have yielded numerous new varieties, but overall the market success of scab resistant cultivars developed to date has been low (Gessler et al., 2006).

Genetic engineering represents a second and faster option for changing domestic apple. Transformation of domestic cultivars occurred in the 1980s and wild apple is a more recent development (James et al., 1989; Zhang et al., 2021). A variety of genetic engineering approaches have been used in apple, including cisgenesis, transgenesis, RNA interference (RNAi), Viral Induced Gene Silencing (VIGS), and Clustered Regularly Interspaced Short Palindromic Repeats (CRISPR, see Table 1 and citations therein). The same apple scab resistance gene introgressed by traditional breeding with *M. floribunda* into domestic apple was transformed directly into Gala apples, maintaining the cultivar background and obtaining resistance in this single generation (Belfanti et al., 2004). Resistance to both apple scab and fire blight occurred with transfer of the *Zea maize* (corn) *Leaf colour* (*Lc*) gene, but the positive gravitropism of the branches meant the trees would be commercially unsuitable for fruit production (Flachowsky et al., 2010a).

**TABLE 1:** Examples of apple traits obtained by genetic engineering. Unless indicated, cultivars are domestic apple.

Cultivar(s)	Trait(s) obtained	Target Gene(s)	Method used	Citation(s)
Royal Gala	Firmer fruit	*MdACS*	RNAi	Hrazdina et al. (2003)
Granny Smith Golden Delicious	Non-browning fruits	*MdPPO*	RNAi	Waltz, (2015)
Galaxy	Anthers converted to petals Reduced pollen and seed formation	*MdMADS15 MdMADS221*	RNAi	Klocko et al. (2016)
Gala	Albino plantlets Early flowering	*MdPDS* *MdTFL1.*1	CRISPR	Charrier et al. (2019)
Golden Delicious Gala	Improved resistance to fire blight	*MdDIPM4*	CRISPR	Pompili et al. (2020)
Fuji Ralls Janet Gala	Improved resistance to *B. dothidea* growth on apple callus	*MdCNGC2*	CRISPR	Zhou et al. (2020)
*Malus sieverii* (wild apple)	Albino plantlets	*MsPDS*	CRISPR	Zhang et al. (2021)
Fuji	Improved resistance to *B. dothidea* fruit rot	*MdCNGC2*	VIGS	Zhou et al. (2020)
Pinova	Columnar tree form	*LEAFY* from *Arabidopsis thaliana*	Transgenesis	Flachowsky et al. (2010a)
Pinova	Early flowering	*MdFT*	Transgenesis	Trankner et al. (2010)
Pinova	Early flowering	*BpMADS4* from *Betula pendula Roth.* (silver birch)	Transgenesis	Flachowsky et al. (2007)
Holsteiner Cox	Increased resistance to fire blight and apple scab	*Leaf colour (Lc)* from *Zea maize*	Transgenesis	Flachowsky et al. (2010b)
Gala	Improved resistance to apple scab	*HcrVF2* from wild apple *Malus floribunda*	Transgenesis	Belfanti et al. (2004)
Gala Galaxy	Improved resistance to fire blight	*FB_MR5* from wild apple *Malus × robusta 5*	Cisgenesis	Kost et al. (2015)
Royal Gala	Yellow fruit	*MdPSY1*	Overexpression	Ampomah-Dwamena et al. (2022)
Holsteiner Cox Gala	Increased phenolics in leaves	*MdMYB10*	Overexpression	Rihani et al. (2017)

Given the long juvenile period of apple trees, one goal of genetic engineering was to produce precocious flowering, which could then be used to shorten generation times and obtain accelerated breeding. This approach required some optimization, as it was challenging to obtain trees that flowered, but not too early. Transgenic addition of *LEAFY* (*LFY*) from *Arabidopsis thaliana* (*Arabidopsis*) led to a more compact and columnar tree form, but no early flowering (Flachowsky et al., 2010b). Overexpression of one *FLOWERING LOCUS T* homolog from domestic apple (*MdFT1*) led to very early flowering with blooms observed while trees were still undergoing *in vitro* cultivation (Trankner et al., 2010). Use of *BpMADS4* from *Betula pendula Roth.* (silver birch) led to the creation of several events, one of which was used to rapidly breed fire blight resistance from an ornamental cultivar of apple to a fruit-bearing cultivar, with five generations occurring in just 7 years (Flachowsky et al., 2007; Schlatholter et al., 2018). This same *BpMADS4* event was also used for introgression of resistance to blue mold from wild *M. sieversii* to Gala (Luo et al., 2020). Individuals with or without the *BpMADS4* gene can be identified by DNA testing, allowing for genotyping in each generation (Luo et al., 2020).

In general, genetic engineering approaches are most straightforward with single genes of large influence, but it is possible to target multiple genes at once in apple with RNAi or CRISPR (Klocko et al., 2016; Jacobson et al., 2023). Unlike their conventionally bred counterparts, engineered trees are subject to many regulations, and are challenging to bring to the commercial market.

### 2.3 Regulatory considerations

Apples and apple products are globally produced and traded, with apples grown in over 90 countries (Nations, 2025). Nearly all apples are conventional crops, produced without genetic engineering, but there is potential for engineered apples to become more prevalent. Currently, non-browning apples obtained by RNAi are grown in Canada and the United States (Waltz, 2015; Duford, 2024). One challenge to wider usage of engineered apples is that regulations regarding definitions and guidelines for genetically engineered crops and crop products are still under development and vary greatly by country and region. Some countries, such as the United States, have guidelines but until recently had no labeling requirements for GMO crops and products. Starting in mid-2025, the United States will now require labeling of foods with ingredients produced by recombinant DNA technology, including Arctic™ apple (Becker, 2023). By contrast, Europe has implemented labeling and monitoring of GMOs, which also included gene-edited crops (Ruffell, 2018). However, a recent decision by the Council of the European Union updated the guidelines to better encompass current plant breeding and modification techniques, which opens up the possibility for future new crop adoptions (Union, 2025). How apple trees such as the fire-blight resistant individuals with the resistance gene introgressed from compatible relatives via genetically accelerated breeding, but which lack transgenes, would be regulated remains to be seen. It is possible that consumers may be in favor of trees produced with these new technologies. There is growing interest in food produced fewer pesticides or fungicides, and if apples produced by new technologies meet consumer needs and desires then regulations may adapt to meet market demand (Rahman et al., 2021).

As science moves quickly, some recent innovations in plant gene targeting offer potential options for achieving transgene-free genome-edited apple trees. It is now possible perform transient CRISPR editing in apple, or to use excision to remove transgenes after edits (Malnoy et al., 2016; Osakabe et al., 2018; Dalla Costa et al., 2020). These approaches, while not highly efficient, are likely to be faster than using breeding to separate transgenes from edits and would allow for maintaining the overall genetic background of the cultivar used. It is possible that if the targeted changes are small and the transgenes are not stably integrated, apple trees produced with this approach could get approval in the European Union or elsewhere. As the field of biotech crops continues to develop, both in terms of science and regulations, it will be interesting to see what is possible, and which possibilities reach consumers. Both growers and consumers may also be a significant influence in the marketability of engineered apples. Transgenic virus-resistant *Carica papaya* (papaya) trees were created and are credited with saving the Hawaiian papaya industry, which at the time was mostly small farmers (Gonsalves, 2006). More recently, the engineered “PinkGlow” *Ananas comosus* (pineapple) was released in the United States as a novelty item and is popular with consumers (Jay, 2024). Perhaps as more engineered fruits enter the global market apples could be part of the portfolio.
