# Supplementary material for: Mini review: Apple improvement, traditional approaches, biotechnology options, and regulatory considerations
Source: Front Bioeng Biotechnol. 2025 Jun 4;13:1617110. doi: 10.3389/fbioe.2025.1617110 (PMC12175005; doi:10.3389/fbioe.2025.1617110)
Supplement: Supplementary file 1 [file DataSheet1.pdf]

Supplementary Material

1 Supplementary Figures and Tables

Supplemental Table 1

Examples of some apple germplasm collections located across the world. This list represents some of the collections and their diversity but is not an exhaustive list of all collections.

Collection location(s)	Brief description of collection	Reference
Bosnia and Herzegovina	165 accessions, domestic apple<br><br>Imported and locally developed cultivars	(Duric, Skytte af Satra et al. 2024)
Brazil	60 cultivars, domestic apple<br><br>Locally developed cultivars	(Mariano, Zchonski et al. 2019)
China	256 cultivars, domestic apple<br><br>Imported and locally developed cultivars	(Tian, Li et al. 2024)
Croatia	169 apple accessions and 11 reference cultivars<br><br>Imported and locally developed cultivars	(Cicek, Konjic et al. 2025)
Czech Republic	273 accessions, domestic apple<br><br>Imported and locally developed cultivars	(Patzak, Paprstein et al. 2012)
Denmark	976 accessions<br><br>Mostly local cultivars	(Larsen, Howard et al. 2025)
France	2,163 accessions, mostly domestic apple<br><br>Heritage and modern cultivars, 2 wild	(Lassois, Denance et al. 2016)
Germany	1,404 cultivars, domestic apple	(Broschewitz, Reim et al. 2024)

	Heritage and modern locally important cultivars	
India	32 genotypes, 31 wild and 1 domestic	(Kumar, Singh et al. 2019)
Iran	33 genotypes, domestic apple<br><br>Local cultivars	(Hassani, Sardoei et al. 2022)
Italy	67 accessions, domestic apple<br><br>Local cultivars	(Alessandri, De Franceschi et al. 2024)
Netherlands	695 accessions, wild and domestic<br><br>Imported and locally developed cultivars	(van Treuren, Kemp et al. 2010)
New Zealand	9,065 trees, wild, domestic, and unknown	(Luby, Alspach et al. 2002)
Norway	345 accessions, wild, domestic, ornamental<br><br>Imported and locally developed cultivars	(Gilpin, Røen et al. 2023)
Norway	181 accessions, domestic apple<br><br>Imported and locally developed cultivars	(Gasi, Kanlic et al. 2016)
Norway	171 accessions, domestic apple<br><br>Local cultivars	(Meland, Aksic et al. 2022)
Romania	34 cultivars, domestic apple<br><br>Imported and locally developed cultivars	(Morariu, Muresan et al. 2025)
Spain	23 cultivars, domestic apple<br><br>Traditional varieties of central Spain	(Arnal, Gogorcena et al. 2020)
Sweden and Finland	101 cultivars, domestic apple<br><br>85 local and 16 imported cultivars	(Garkava-Gustavsson, Mujaju et al. 2013)

Turkey	212 cultivars, domestic apple<br><br>206 local and 6 reference varieties	(Bakir, Dumanoglu et al. 2022)
Ukraine	139 cultivars, domestic apple<br><br>Imported and locally developed cultivars	(Udovychenko, Kondratenko et al. 2024)
United States of America	101 accessions, wild *Malus orientalis*	(Volk, Richards et al. 2009)

Supplementary References

Alessandri, S., P. De Franceschi, N. Alvisi and L. Dondini (2024). "Determination of S-Allele Combination in an Italian Apple (*Malus* × *domestica* Borkh.) Germplasm Core Collection." Plant Breeding **0**: 1-8.

Arnal, A., Y. Gogorcena, J. Tardio, I. Roldan-Ruiz and A. Lazaro (2020). "Simple Sequence Repeat Characterisation of Traditional Apple Cultivars (Malus domestica Borkh.) Grown in the Region of Madrid (Central Spain)." Plant Molecular Biology Reporter **38**: 676-690.

Bakir, M., H. Dumanoglu, A. Aygun, V. Erdogan, S. E. Dost, O. Gulsen, U. Serdar, O. Kalkisim and K. Bastas (2022). "Genetic diversity and population structure of apple germplasm from Eastern Black Sea region of Turkey by SSRs." Scientia Horticulturae **294**: 110793.

Broschewitz, L., S. Reim, H. Flachowsky and M. Hofer (2024). "Pomological and Molecular Characterization of Apple Cultivars in the German Fruit Genebank." Plants (Basel) **13**(19).

Cicek, D., A. Konjic, M. Skendrovic Babojelic, P. Vujevic, S. Simon and F. Gasi (2025). "Genetic Uniqueness and Pomological Diversity Among the Apple Accessions MaintainedWithin the Croatian National Clonal Germplasm Repository." Agronomy **15**: 113.

Duric, G., J. Skytte af Satra, F. Gasi, A. Konjic, H. Flachowsky, N. P. Howard, M. Kajut Zeljkovic and L. Garkava-Gustavsson (2024). "Genetic diversity of apple heirloom germplasm in Bosnia and Herzegovina, as revealed by SNP markers." Tree Genetics & Genomes **20**: 28.

Garkava-Gustavsson, L., C. Mujaju, J. Sehic, A. Zborowska, G. M. Backes, T. Hietaranta and K. Antonius (2013). "Genetic diversity in Swedish and Finnish heirloom apple cultivars revealed with SSR markers." Scientia Horticulturae **162**: 43-48.

Gasi, F., K. Kanlic, B. K. Stroil, N. Pojskic, A. Asdal, M. Rasmussen, C. Kaiser and M. Meland (2016). "Redundancies and Genetic Structure among ex situ Apple Collections in Norway Examined with Microsatellite Markers." HortScience **51**: 1458-1462.

Gilpin, L., D. Røen, M. Schubert, J. Davik, K. Rumpunen, K. A. Gardli, S. H. Hjeltnes and M. Alsheikh (2023). "Genetic Characterization of the Norwegian Apple Collection." Horticulturae **9**: 575.

Hassani, S. A., A. S. Sardoei, H. A. G. Bigloo, H. Ghasemi and A. Ghorbanzadeh (2022). "Assessment of Genetic Diversity in Iranian Apple Genotypes Using SSR Markers." International Journal of Horticultural Science and Technology **4**: 487-496.

Kumar, C., S. K. Singh, R. Singh, K. K. Pramanick, M. K. Verma, M. Srivastav, G. Tiwari and D. R. Choudhury (2019). "Genetic diversity and population structure analysis of wild *Malus* genotypes including the crabapples (*M. baccata* (L.) Borkh. & *M. sikkimensis* (Wenzig) Koehne ex C. Schneider) collected from the Indian Himalayan region using microsatellite markers." Genetic Resources and Crop Evolution **66**: 1311-1326.

Larsen, B., N. P. Howard, C. Denance, C. E. Durel, C. Pedersen, J. Skytte af Satra, L. Garkava-Gustavsson, M. Troggio and E. van de Weg (2025). "Cultivar fingerprinting and SNP-based pedigree reconstruction in Danish heritage apple cultivars utilizing genotypic data from multiple germplasm collections in the world." Genetic Resources and Crop Evolution **72**: 2397-2411.

Lassois, L., C. Denance, E. Ravon, A. Guyader, R. Guisnel, L. Hibrand-Saint-Oyant, C. Poncet, P. Lassere-Zuber, L. Feugey and C.-E. Durel (2016). "Genetic Diversity, Population Structure, Parentage Analysis, and Construction of Core Collections in the French Apple Germplasm Based on SSR Markers." Plant Molecular Biology Reporter.

Luby, J. J., P. A. Alspach, V. Bus and N. C. Oraguzie (2002). "Field Resistance to Fire Blight in a Diverse Apple (*Malus* sp.) Germplasm Collection." Journal of the American Society for Horticultural Science **127**: 245-253.

Mariano, L. C., F. L. Zchonski, C. M. da Silva and P. R. Da-Silva (2019). "Genetic variability in a Brazilian apple germplasm collection with low chilling requirements." Peer J **6**: e6265.

Meland, M., M. F. Aksic, O. Froynes, A. Konjic, L. Lasic and N. Pojskic (2022). "Genetic Identity and Diversity of Apple Accessions within a Candidate Collection for the Norwegian National Clonal Germplasm Repository." Horticulturae **8**: 630.

Morariu, P. A., A. E. Muresan, A. F. Sestras, A. E. Tanislav, C. Dan, E. Maresi, M. Militaru, M. V. and R. E. Sestras (2025). "A Comprehensive Morphological, Biochemical, and Sensory Study of Traditional and Modern Apple Cultivars." Horticulturae **11**: 264.

Patzak, J., F. Paprstein, A. Henychova and J. Sedlak (2012). "Comparison of genetic diversity structure analyses of SSR molecular marker data within apple (Malusxdomestica) genetic resources." Genome **55**(9): 647-665.

Tian, W., Z. Li, L. Wang, S. Sun, D. Wang, K. Wang, G. Wang, Z. Liu, X. Lu, J. Feng and Y. Gao (2024). "Comprehensive Evaluation of Apple Germplasm Genetic Diversity on the Basis of 26 Phenotypic Traits." Agronomy **14**: 1264.

Udovychenko, K., T. Kondratenko, K. Tarnavska, V. Voloshyna, J. Skytte af SÃ¤tra and L. Garkava-Gustavsson (2024). "Genetic diversity of Ukrainian apple germplasm estimated with SSR markers." Acta Horticulturae **1412**: 69-74.

van Treuren, R., H. Kemp, G. Ernsting, B. Jongejans, H. Houtman and L. Visser (2010). "Microsatellite genotyping of apple (*Malus* x *domestica* Borkh.) genetic resources in the Netherlands:

application in collection management and variety identification." Genetic Resources and Crop Evolution **57**: 853-865.

Volk, G. M., C. M. Richards, A. D. Henk, A. A. Reille and P. A. Reeves (2009). "Capturing the Diversity of Wild *Malus orientalis* from Georgia, Armenia, Russia, and Turkey." Journal of the American Society for Horticultural Science **134**: 453-459.
